# Supplementary material for: Complexome profiling of the Chlamydomonas psb28 mutant reveals TEF5 as an early PSII assembly factor
Source: Plant Cell. 2025 Mar 17;37(6):koaf055. doi: 10.1093/plcell/koaf055 (PMC12164590; doi:10.1093/plcell/koaf055)
Supplement: koaf055_Supplementary_Data [file koaf055_supplementary_data.zip › psb28_interactive.zip!/plots/intensity/qualityPlots/peptides/ELI4_Cre02.g143550.t1.2.html]

### Cre02.g143550.t1.2

Trivial name: ELI4  
SeqCoverage [median]: 13.60  
Mass: 29.81 kDa  
  
 

| Sequence | MissedCleavage | Mass [Da] | wtIntensity | muIntensity | Score |
| --- | --- | --- | --- | --- | --- |
| ADSAAAELINGR | 0 | 1186.59 | 0.00 | 1887.12 | 125.48 |
| ATLLELTGIPLQK | 0 | 1395.83 | 1020.66 | 0.00 | 117.67 |
| FPAVFGPDNK | 0 | 1090.54 | 415.46 | 7679.85 | 110.87 |
| GVRFPAVFGPDNK | 1 | 1402.74 | 0.00 | 4187.79 | 40.73 |
|  |  |  | 1436.1 | 13754.8 |  |
